# The Impact of the Medical Insurance System on the Health of Older Adults in Urban China: Analysis Based on Three-Period Panel Data

**DOI:** 10.3390/ijerph20053817

**Published:** 2023-02-21

**Authors:** Hongfeng Zhang, Peng Cheng, Lu Huang

**Affiliations:** 1 School of Public Administration and Policy, Shandong University of Finance and Economics, Jinan 250014, China; 2 School of Economics, Shandong University of Finance and Economics, Jinan 250014, China

**Keywords:** social medical insurance, commercial medical insurance, health, older adults, China, wellness and health promotion

## Abstract

The impact of the medical insurance system (MIS) on the health of older adults is a key element of research in the field of social security. Because China’s MIS consists of different types of insurance, and the benefits and levels of coverage received by participating in different medical insurance vary, different medical insurance may have a differential impact on the health of older adults. This has rarely been studied before. In this paper, the panel data of the third phase of the China Health and Retirement Longitudinal Study (CHARLS) conducted in 2013, 2015 and 2018 were used to investigate the impact of participation in social medical insurance (SMI) and commercial medical insurance (CMI) on the health of urban older adults and its mechanism relationship. The study found that SMI had a positive impact on the mental health of older adults, but only in the eastern region. Participation in CMI was positively correlated with the health of older adults, but this association was relatively small and was only observed in the sample of older adults aged 75 years and above. In addition, future life security plays an important role in the process of improving the health of older adults through medical insurance. Both research hypothesis 1 and research hypothesis 2 were verified. The results of this paper show that the evidence of the positive effect of medical insurance on the health of older adults in urban areas proposed by scholars is not convincing enough. Therefore, the medical insurance scheme should be reformed, focusing not only on coverage, but on enhancing the benefits and level of insurance, so as to enhance its positive impact on the health of older adults.

## 1. Introduction

Health is an inevitable requirement for the promotion of all-round human development and is a common aspiration of people all over the world. The 2021 data from the 7th National Census shows that China’s population aged 60 and above is 260 million, an increase of 5.4% over the 6th National Census data. According to a survey conducted by the China Health and Wellness Commission, the percentage of older adults suffering from chronic diseases is as high as 75%. It follows that the number and health status of older adults have changed. In order to reduce the risk to the health of older adults due to disease, the establishment of a comprehensive medical insurance system (MIS) has become an important policy challenge for countries around the world. By the end of 2021, the number of people participating in social medical insurance (SMI) in China has exceeded 1.3 billion, with the participation rate stable at over 95%. The closer the participation rate of SMI is to universal coverage, which has a catalytic effect on reducing the psychological burden of patients and increasing the utilization of medical services [1,2], is also crucial to economic and social development [3]. However, the relationship between SMI coverage and health is more controversial, with some studies suggesting that participation in SMI is beneficial to health improvement [4,5] and others showing that expansion of SMI coverage does not necessarily improve health [6,7].

China has continued to improve the multi-level medical security system with basic medical insurance as the main body, supplemented by other various forms of supplementary insurance and commercial medical insurance (CMI), to gradually improve the level of protection and service capacity of commercial insurance, and provide systematic health services for the people. The total income of CMI premiums increased from 2.8 billion yuan in 2000 to 880.36 billion yuan in 2021, an increase of nearly 320 times. This fully reflects the importance that the Chinese government attaches to CMI and shows that CMI is of great value in meeting the multi-level health needs of the people and improving their health. As an important supplement to basic medical insurance, CMI can effectively play the role of improving social welfare and relieving the pressure of medical treatment [8]. CMI has been verified by scholars at home and abroad for reducing the risk of illness among older adults and improving the health of the insured group [9,10,11].

There are disparities between SMI and CMI groups in terms of coverage benefits, levels and medical services [12], which has led to health variability among different medical insurance groups. Existing studies have explored many factors influencing the health of older adults, but examining the role of medical insurance on the health of older adults lacks sufficient attention, especially the health status of different types of insurance groups, which has not received sufficient attention. This is an important issue in the transformation of China’s MIS reform from a focus on coverage improvement to diversity and equity.

Scholars have performed a great deal of analysis on the impact of the MIS on the health of older adults, but more often than not, the impact of a particular medical insurance on the health of older adults has been studied separately. Meanwhile, the evaluation of the health of older adults is mostly based on the ability of daily activities and cognitive ability, and the measurement index is relatively single. The health status of older adults needs to be measured comprehensively from multiple aspects. In view of this, this study analyzes the impact of the MIS on the health of older adults in urban areas based on the China Health and Retirement Longitudinal Study (CHARLS) three-period panel data, and further investigates the heterogeneity and transmission mechanism of this impact. Based on this, the marginal contribution of this paper is mainly reflected in the following three aspects. First, consider the impact of different types of medical insurance on the health of older adults in urban areas. This paper discusses the health status of older adults in urban areas who participate in SMI and CMI, and analyzes how different types of medical insurance affect the health status of older adults in urban areas, breaking through the limitations of existing studies which only focus on the coverage of medical insurance. Second, consider the path of medical insurance. Medical insurance can effectively play the role of spiritual security, and participation in medical insurance can improve residents’ sense of security and future life security. Therefore, this paper analyzes the internal mechanism of future life security in medical insurance affecting the health of older adults in urban areas. Thirdly, the influence of demographic characteristics and regional characteristics is considered comprehensively. This paper analyzes the age and regional heterogeneity of the influence of medical insurance on the health of older adults in urban areas, which is helpful to grasp the practical operation of medical insurance and deepen the internal mechanism of medical insurance. By comparing the effects of SMI and CMI on the health of older adults in urban areas, it can provide a theoretical basis for the adjustment and improvement of medical insurance policies.

## 2. Theoretical Analysis Framework

### 2.1. Definition Study of Health

Health is a state in which a person is in good physical, mental, and social condition. In 1989, the World Health Organization proposed that health is not only the absence of disease in the body, but also a state of mental health and good social adaptation, in which individuals can perform their daily tasks positively and effectively and maintain a good state of mind and character. Some scholars believe that health is not only the health of people’s psychological world, and the study of health should be directed to people’s overall situation [13]. Other scholars point out that health is a good state of social life in which individuals can actively engage in social participation, fully develop their psychological potential, and have good self-perception [14,15]. With the increase of aging and the rise of the disease rate in the older adults group, the health status of older adults presents different characteristics [16]. Scholars have defined the health status of older adults mainly in three aspects: physical health, mental health and self-health [7,13].

### 2.2. Impact of the MIS on the Health of Older Adults

In order to improve the health level of older adults and ensure that they enjoy equalized medical and health services, governments around the world have introduced many policy measures, and the Chinese government has also continued to establish and improve the MIS and medical service system with Chinese characteristics, which is of great significance in improving the health status of older adults and providing health services throughout their life cycle.

Different MIS are a manifestation of the interaction of economic and social factors, and the selection of an appropriate MIS has an important impact on the health status of older adults. This paper mainly analyzes the impact and mechanism of different types of MIS on the health of older adults. SMI is enforced by the government through legislation to establish an SMI fund, and workers are given appropriate subsidies or reimbursement when they fall ill [17]. It has been argued that participation in SMI can improve the financial access to health care and the accessibility of health care services for older adults [18,19,20], which somehow increases the likelihood of maintaining good health in older adults groups. This is because SMI shares the financial risk of the sick group and guarantees the utilization of medical and health services. CMI is a voluntary insurance for units and individuals, and the insurance premiums paid are operated by insurance agencies, from which they can receive a certain amount of medical expenses in case of major diseases [21]. Related studies have shown that cognitive ability, mental health, and self-health of older adults who participate in CMI are improved to a greater extent [22,23,24,25], and will improve the health of participants through the channels of increased financial accessibility to medical care, reduced precautionary savings, and improved lifestyles. It has also been noted that medical insurance does not positively affect the health status of older adults [26,27]. It has been demonstrated in different ways that the MIS has a significant effect on the health of older adults, but there are many factors that influence the health of older adults, such as the type of disease, economic income, and living environment, and the health-promoting effect of medical insurance may be reduced or even disappear [28,29]. The health role of medical insurance may or may not be present [30,31]. Therefore, more in-depth research is needed to determine whether medical insurance is effective in improving the health of older adults. Based on the above analysis, the following research hypothesis is proposed in this paper.

 **Hypothesis 1a (H1a).** Medical insurance has a significant impact on the health of older adults.

 **Hypothesis 1b (H1b).** Medical insurance has no impact on the health of older adults.

### 2.3. The Role of Mediating: Future Life Security

The health of older adults is multidimensional and influenced by a variety of factors and pathways. Medical insurance functions as a financial accessibility and economic risk sharing for medical care, which to some extent reduces the economic risk of the sick group and enhances the utilization of medical services for the insured individuals [2]. Thus, it can be seen that medical insurance plays a significant role in material security and economic security. Medical insurance has a significant impact effect on the health of older adults, and there may be other important effects in addition to the health effects from medical service utilization that have been widely validated. The group enrolled in medical insurance has better self-soothing ability [32], which facilitates the function of spiritual security and subjective comfort and enhances their sense of security for their future life.

As an effective protection mechanism for patients to cope with health risks, medical insurance plays an important role in enhancing the sense of security and confidence in the future life of the insured group [33]. Some scholars argue that individuals who participate in medical insurance have a greater sense of well-being and security and a higher level of health compared to individuals who do not participate in medical insurance [34]. It has also been noted that the group with medical insurance has a good sense of security and health, and they have a higher happiness index in life [35,36]. In case of illness, medical insurance can reimburse medical expenses, which reduces the psychological pressure of the participants, and medical insurance can also provide price subsidies for the participants, which reduces the possibility of “not getting medical treatment” and increases the participants’ hope for their future life. However, there are differences in the medical resources available to individuals with different MIS, and these medical resources are an important factor in future life security. Therefore, it is reasonable to suggest that MIS can influence the health status of older adults by affecting their future life security. In view of the above analysis, the following research hypothesis is proposed in this paper.

 **Hypothesis 2 (H2).** Future life security plays a mediating role in the impact of the medical insurance on the health of older adults, but the degree of influence may vary across dimensions of health.

**Figure 1:**
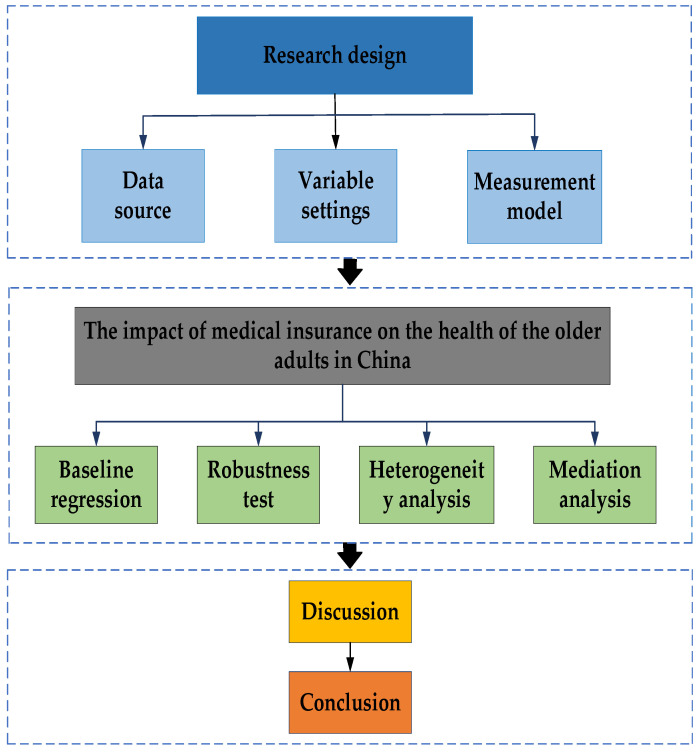
Analytical framework of China’s MIS affecting the health of urban older adults.

Finally, based on the literature analysis and research hypotheses, this paper proposes a corresponding analytical framework for medical insurance reform and health improvement of urban older adults in China (as shown in Figure 1).

## 3. Materials and Methods

### 3.1. Data

This paper uses data from the CHARLS organized and implemented by the China Social Science Survey Center of Peking University in 2013, 2015 and 2018, which used a scientific sampling method to select 150 counties in proportion to their probability, and each county then randomly selected three villages or communities, and each village or community then randomly selected people over 45 years old as the main respondents, collecting about 20,000 valid. The sample size is wide and representative. The data provide basic information, family information, and work status of older adults, including their insurance status and health status. In this paper, we mainly select respondents who are urban residents and are sixty and above for statistical analysis, excluding samples below sixty. During the study, we excluded the samples with missing key variables in the data, but it did not affect the validity of statistical inference, and finally collated 2403 valid samples.

### 3.2. Description of Variables

#### 3.2.1. Dependent Variable

The dependent variable is the health of older adults in urban areas. In this paper, the health status of older adults was measured in several dimensions, mainly including physical health, mental health and self-health of older adults. In terms of physical health, it is mainly based on the Activities of Daily Living (ADL) scale, which measures six items in the questionnaire: dressing, eating, bathing, toileting, transferring out of bed, and doing household activities, and the respondents’ scores are processed in reverse order, and the range of values is 6 to 24, with higher scores indicating better physical health. In terms of mental health, the questionnaire was used to determine the frequency of 10 situations in the last week, including “feeling depressed, struggling to do anything, not sleeping well, feeling lonely, worrying about small things, feeling unable to continue living, having difficulty concentrating, feeling happy, etc. “The options “don’t know” and “refused to answer” were eliminated from the questionnaire. The scores of negative emotions were reversed, 1 to 4 for most of the time, sometimes or half of the time, not too much, rarely or not at all, and 1 to 4 for positive emotion, respectively, according to the questionnaire. In terms of self-assessment of health, the questionnaire was based on the question “How do you think your health is?” Respondents’ scores were processed in reverse order, and the options were assigned as 1 = very poor, 2 = poor, 3 = fair, 4 = good, and 5 = very good, with higher scores indicating better self-health.

#### 3.2.2. Independent Variable

The independent variable is the MIS. The MIS includes SMI and CMI. SMI is measured according to the questionnaire “Are you currently participating in urban workers’ medical insurance, urban and rural residents’ medical insurance”, as long as the respondent participates in one of the medical insurances, he/she is considered to have SMI and is assigned a value of 1, otherwise, he/she is assigned a value of 0. CMI is measured by the questionnaire “Do you participate in CMI purchased by your organization or individually”, and respondents are considered to have CMI as long as they participate in one of the CMI and are assigned a value of 1, otherwise they are assigned a value of 0. Since respondents can only participate in one of the MIS, therefore, the two variables add up to 1.

#### 3.2.3. Control Variables

This paper controls for individual characteristics of older adults and variables that may affect individual health, which include three main categories. The first category: demographic characteristics variables. They mainly include gender, age, education level, and marital status. “Married living with spouse” and “married, but not living with spouse temporarily because of work and other reasons” were combined into “normal marriage” and assigned a value of 1. “Separated”, “divorced”, “widowed”, “never married” are combined as “The second category: socio-economic characteristics variables. It mainly consists of the number of children and pension insurance. The number of children was based on the questionnaire “How many children do you have?” Pension insurance is measured according to the questionnaire: “Are you currently participating in/receiving urban workers’ pension insurance, supplementary pension insurance or urban and rural residents’ pension insurance? Respondents are considered to have pension insurance as long as they participate in one of the pension insurances, and are assigned a value of 1. Otherwise, they are assigned a value of 0. The third category: health characteristics variables. The influence of chronic diseases and health behaviors is mainly examined. Chronic diseases were measured according to the questionnaire “Have any doctors ever told you that you have chronic diseases such as hypertension, diabetes and kidney disease?” was measured. If the respondent suffers from one of the chronic diseases, it will be set as a chronic disease patient, with a value of 1, otherwise it will be 0. Health behavior was measured based on the questionnaire “Do you ride a bicycle, play tai chi, walk, play sports, exercise, walk, etc. every week? was measured. Respondents were assigned a value of 1 if they answered yes, and 0 otherwise.

#### 3.2.4. Mediating Variable

**Figure 2:**
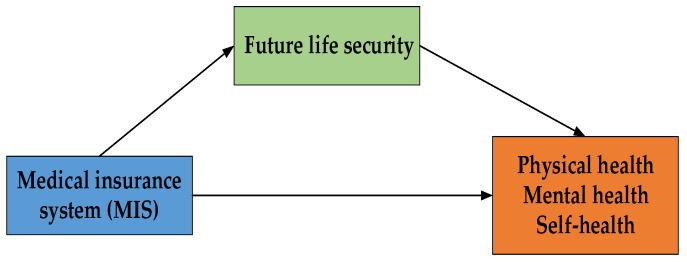
Mechanism of action diagram.

The mediating variable is future life security. In addition to analyzing the direct impact of the MIS on the health of older adults, this paper also examines whether the MIS affects the health of older adults through other channels. The security of future life is measured by the questionnaire “Have you been hopeful about the future in the past week”. The mediating variable was a continuous variable. Figure 2 shows the mechanism of the effect of medical insurance on the health of older adults in urban areas in China.

### 3.3. Model Description

#### 3.3.1. OLS Model

Since the dependent variables in this paper are continuous variables, a multiple linear regression model is used to estimate the effect of the MIS on the health of older adults. The model is expressed in the following equation.

(1) $$H_{i}=\alpha +\beta X_{i}+\gamma Z_{i}+\varepsilon_{i}$$

In the formula, $H_{i}$ represents the health status of older adults (physical health, mental health, and self-health), $X_{i}$ represents the medical insurance status of older adults (SMI, CMI), and $Z_{i}$ represents the control variables (demographic characteristics, socioeconomic characteristics, and health characteristics); α, β, and γ represent the parameters to be estimated; and $\varepsilon_{i}$ represents the random disturbance terms, including elements that are not considered in this study but affect the health of older adults.

#### 3.3.2. Mediating Effect Model

According to the previous theoretical analysis, the main mechanism of the MIS’s influence on the health of older adults is future life security. In order to test the role of future life security in the MIS’s influence on the health of older adults, this study uses the mediating effect model to test it. The model was set up with the following equation.

(2) $$H_{i}=\alpha +\beta X_{i}+\gamma Z_{i}+\varepsilon_{i}$$

(3) $$M_{i}=\varphi_{0}+aX_{i}+\gamma Z_{i}+\varepsilon_{i}$$

(4) $$H_{i}=\varphi_{1}+c\beta X_{i}+bM_{i}+\delta Z_{i}+\sigma_{i}$$

Equation (2) represents the total effect of the MIS on the health of older adults; Equation (3) represents the effect of the MIS on the mediating variables; and Equation (4) represents the effect of the MIS on the health of older adults after adding the mediating variables. *a*, *b*, and *c* are significant, indicating that there is a partial mediating effect; *a* and *b* are significant but *c* is not, indicating that there is a full mediating effect; and when at least one of *a* and *b* insignificant and *c* significant, then a Sobel test is required and if significant, then there is a mediating effect.

## 4. Results

### 4.1. Descriptive Statistical Analysis

#### 4.1.1. Basic Characteristics of Older Adults

According to the sample data of this study, the mean values of physical health, mental health and self-health of the urban older adults are 22.97, 33.61 and 3.13, respectively, which shows that there is still more room for improving the health level of urban older adults in all dimensions. The proportions of urban older adults with SMI and CMI were 89.19% and 35.15%, respectively. The average age of the study sample was 70.28 years old, and the mean value of education level was 1.89, mainly concentrated in primary and junior high school education, and the proportion of females in the sample was 52.71%. The proportion of the sample with normal marriages was 79.01%, which indicates the relatively harmonious marriages of urban older adults. The proportion of urban older adults with chronic diseases is 27.62%, which shows that urban older adults are less likely to suffer from chronic diseases. The proportion of urban older adults who have pension insurance is 75.69%. The proportion of urban elderly who have health behaviors is 91.18%. For details, see Table 1.

#### 4.1.2. Inequality in Medical Insurance Types and the Health of Older Adults

**Figure 3:**
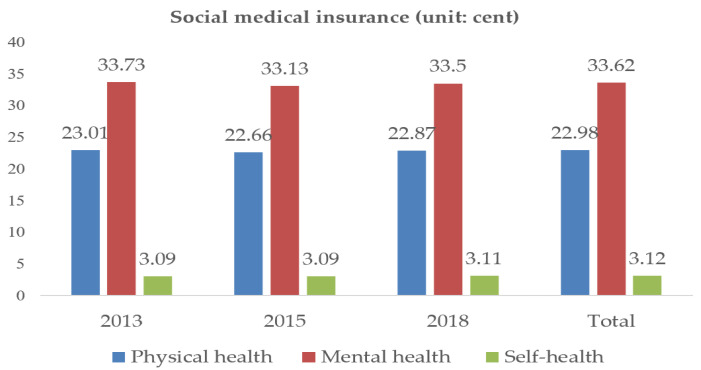
Health status of social medical insurance groups.

**Figure 4:**
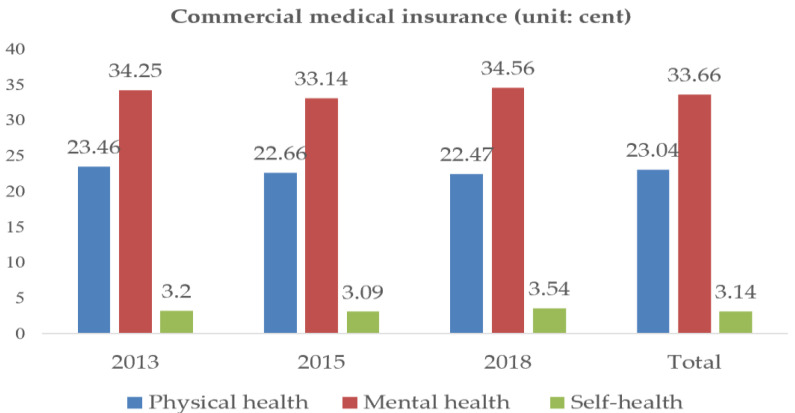
Health status of commercial medical insurance groups.

Separate measurement of different dimensions of the health of urban older adults allows us to clearly see the physical health, mental health, and self-health status of urban older adults who participate in different types of medical insurance. Figure 3 and Figure 4 provides an overview of the health status of the total sample and sub-sample participant groups.

Overall, the mean physical health, mental health, and self-health values of urban older adults participating in CMI were higher than those of urban older adults participating in SMI, by 0.06, 0.04, and 0.02 points, respectively. By year: in 2013, the health status of urban older adults who participated in CMI and SMI was basically the same as the total sample; in 2015, the health status of the sample data of those who participated in SMI and CMI was basically the same, with the same physical health and self-health scores; in 2018, the mental health and self-health of urban older adults who participated in CMI were higher than those who participated in SMI, with 1.06 and 0.43 points higher, respectively. The above analysis reflects that the health level of the urban older adults participating in CMI is higher than that of those participating in SMI, the high coverage rate of basic medical insurance does not represent the good health status of the urban older adults.

### 4.2. Regression Analysis of the MIS on the Health of Older Adults

Table 2 shows the results of the multiple linear regression of medical insurance in China. To determine the effects of different types of medical insurance on the health status of older adults, model 2-1 classified medical insurance into two types: SMI and CMI. The results showed that the health status of older adults with SMI and CMI did not improve significantly compared to those without medical insurance. However, the extent of the positive effect of CMI on the health of older adults was greater compared to the extent of the effect of SMI on the health of older adults.

Model 2-2 was based on model 2-1 by adding control variables such as demographic characteristics, socioeconomic characteristics and health behavior characteristics. The results from the medical insurance regression model 2-2 showed that when other control variables were added, the effects of SMI and CMI on the health of older adults remained largely consistent with model 2-1, no significant effects were produced. H1b was tested.

Chronic variables had a significant negative effect on different dimensions of health of older adults. The possibility of poorer health status is higher in older adults with chronic diseases, which is because having chronic diseases such as hypertension and chronic bronchitis affects the daily life and organism activities of older adults, which in turn negatively affects their physical health; at the same time, older adults are more likely to judge the severity of the disease by subjectively felt symptoms, which often increases the negative psychological implication of older adults, leading to self-health and negative evaluations of mental health. Health behavior variables had a significant positive effect on different dimensions of health status of older adults, older adults with health behaviors such as walking, exercise, and sports had better health status. This may be due to the following reasons: health behaviors can reduce loneliness and depression of older adults, prevent the decline of self-care ability and instrumental daily living ability in older adults, increase life satisfaction, and thus improve their physical health and mental health; older adults can participate in health behaviors, their own subjective perception of relatively better health, and a relatively high evaluation of self-health. The number of children has a significant negative effect on the physical and mental health of older adults. On the one hand, it is because older adults’ physical health is adversely affected by taking care of a large number of children in their younger years, and the large number of children increases the intensity and frequency of intergenerational care, and intergenerational care in old age further damages their physical health, which in turn has a negative impact on the physical health of older adults; on the other hand, it is because a large number of children will increase family chores, and older adults worry about their children’s work, family, health, etc., they will have various psychological disorders and worries, which will affect their mental health. The marriage variable has a significant positive effect on the mental health of older adults, probably because a harmonious marriage is conducive to the maintenance of a good lifestyle and a positive attitude toward life, which is conducive to spiritual cultivation and soul enhancement, and thus can have a positive effect on the mental health of older adults.

### 4.3. Robustness Tests

In this paper, from the perspective of replacing the econometric model and sample data, the robustness test of the underlying regression results is conducted by using a panel fixed effects model and new sample data. The results of the analysis using the panel fixed effects model and new sample data are shown in Table 3. From Table 3, it can be seen that the coefficients of the independent variables, SMI and CMI, are not significant, indicating that medical insurance does not promote the health of older adults in urban China, and the results of the base regression are verified. For the other control variables, the positive effects of health behaviors passed the significance test, and the results of the underlying regression were verified.

### 4.4. Heterogeneity Analysis

#### 4.4.1. Regional Heterogeneity Test

Considering that the eastern, central and western regions of China are quite different in terms of economic development level, infrastructure construction, social welfare treatment, etc. Therefore, in reference to existing research [37], according to the division method of China National Bureau of Statistics, this paper will divide the province (district, city) where the sample data is located into three sub sample data regions, namely, the east, the central, and the west, to investigate the impact of the MIS in different regions on the health of urban older adults. It can be seen from the estimation results in Table 4 that the estimation results in the western and central regions are basically consistent with the basic regression results in Table 2. CMI and SMI have no significant impact on the health of older adults at the statistical level, while CMI in the eastern region has no significant impact on the health of urban older adults at the statistical level. Only SMI has a significant impact on the mental health of older adults at the statistical level. H1a was tested.

#### 4.4.2. Age Heterogeneity Test

Groups with different demographic characteristics have different health status, and the number of medical services used by different groups will be affected by age factors. Therefore, in this paper, the sample data will be divided into two sub-sample data of older adults between age 60 and 74 and older adults aged 75 and above according to the age division criteria, and the impact of medical insurance on the health of urban older adults in different age groups will be examined separately. From the estimation results in Table 5, it can be seen that the estimated results of older adults aged 60 and 74 are basically consistent with the results of the base regression in Table 2, which indicates that both CMI and SMI have no statistically significant effects on the health of the old adults at the statistical level. In contrast, participation in CMI has a statistically significant effect on the health of older adults aged 75 and above at the statistical level. H1a was tested again.

### 4.5. Further Study: Mediating Effects

The heterogeneity analysis above verified that CMI has a significant effect on the health of older adults aged 75 and above, and SMI has a significant effect on the mental health of older adults living in eastern China. This paper next verifies the mediators of the effect of CMI on the health of older adults aged 75 and above and the mediators of the effect of SMI on the mental health of older adults living in eastern China. We used the three-step regression method to estimate the mediating effect of future life security and control for covariates.

#### 4.5.1. Regression Results of Mediating Effects in the Effect of CMI on the Health of Older Adults Aged 75 and Above

The results of the mediating regression of future life security in the effect of CMI on the health of older adults are shown in Table 6. The coefficients of the mediating variable future life security all passed the test at the 5% statistical level, indicating that CMI effectively improves the health status of older adults by significantly enhancing their future life security. Specifically, models 6-1, 6-4, and 6-7 are the main effects of CMI on the health of older adults, and the other models in Table 6 are tests of the mediating effects of future life security. The main effects regression results show that the coefficient of CMI is positive at the 10% significance level, indicating that CMI significantly enhances the health of older adults aged 75 and above. The results of the mediating effect regression showed that future life security played a positive mediating role in the improvement of health of older adults aged 75 and above by CMI, and the proportion of the mediating effect to the total effect was 13.594%, 31.959%, and 28.654%, respectively. Therefore, H2 was verified.

#### 4.5.2. Regression Results of Mediating Effects in the Effect of SMI on the Mental Health of Older Adults Living in Eastern China

The mediating effect model was constructed to empirically test the mechanism of the role of future life security in SMI on the mental health of older adults living in eastern China, and the regression results are shown in Table 7. Model 7-1 shows the main effect of SMI on the mental health of older adults living in eastern China, and Model 7-2 and Model 7-3 show the mediating effect of future life security. The main effect regression results show is that the coefficient of SMI is positive at the 5% level of significance, indicating that SMI significantly enhances the mental health of older adults living in eastern China. The regression results of the mediating effect showed that future life security played a positive mediating role in SMI to enhance the mental health of older adults living in eastern China, and the proportion of the mediating effect to the total effect was 19.583%. Therefore, SMI can effectively improve the health of older adults living in eastern China by increasing their sense of future life security. H2 was again verified.

## 5. Discussion

We investigated the relationship between different MIS and the health of older adults in urban China. Our longitudinal regression analysis based on phase III panel data shows that SMI has a positive impact on the mental health of older adults, but only in specific areas. Participation in CMI is positively related to the health of older adults, but this association is relatively small, which is only observed in older adults aged 75 and above. This shows that the coverage rate of medical insurance is not positively related to the health of urban older adult groups, and the current plan of full coverage of medical insurance has research limitations. These research results are basically consistent with those of Chinese scholar Yu Da chuan [33,38]. As for the relationship between medical insurance and the health of older adults, our results are in sharp contrast with those of Cheng Ling guo [39], who reported more positive results using Chinese Longitudinal Healthy Longevity Survey (CLHLS) data. However, our research is different from theirs. We used the data of the three longitudinal surveys from 2013 to 2018, while they only used the data of two periods (2005–2008). As for the relationship between China’s medical insurance and the health of older adults, most previous studies have reported that the groups participating in medical insurance have good health conditions. These studies use data earlier than our study (2013–2018) (such as 2005–2011), which shows that the impact of medical insurance on the health of older adults should not only focus on short-term effects.

We examined the mediating role of future life security. The results show that the MIS can have a positive and indirect impact on the health of urban older adults groups by increasing the future life safety of the insured groups. As we all know, with the growth of age, the health status of older adults will inevitably decline, and the probability of illness will also increase, which hinders the possibility of older adults to enjoy a happy old age. In order to mitigate the negative impact and consequences of the disease, they often obtain a health risk protection mechanism by participating in medical insurance, reduce the concern of the insured group about the economic burden of the disease, and enhance their sense of security in future life, so as to maintain a good health level. Although some scholars pointed out that medical insurance has little effect on health, the improvement of the health level of older adults in China still requires medical insurance to play a role [40].

In this study, control variables such as health behavior and chronic diseases have significant effects on the health of urban older adults. The urban older adults with healthy behaviors were more likely to be in good health [41,42,43], because their physique was improved and their mood was relaxed through exercise, walking and other healthy behaviors, which is conducive to improving the health of older adults. In addition, urban older adults without chronic diseases are more likely to be in good health. Datta et al. found that older adults without chronic diseases had good health in all aspects [44].

According to the results of this study, the Chinese government should increase medical insurance benefits to effectively provide medical resources for urban older adult groups, especially urban low-income groups. The government should also reform the MIS. Against the background that medical insurance is close to full coverage, it should reduce the medical insurance costs of urban residents, and cover a wider range of outpatient patients and more types of diseases.

## 6. Conclusions

Through the above research, the following conclusions are drawn: firstly, the promotion effect of CMI on the health of older adults is greater than that of SMI. As shown in Figure 3, the health level of older adults participating in CMI is higher than that of older adults participating in SMI; secondly, SMI has a positive impact on the mental health of older adults, but only in certain areas. CMI was positively correlated with the health of older adults, but this association was relatively small and was only observed in the sample of older adults aged 75 years and above; thirdly, medical insurance is greatly influenced by future life security in improving the health level of older adults in urban areas. Medicare improves the health of older adults by increasing their future security. The policy implications of the above conclusions are as follows: first, the MIS plays a certain role in promoting the health of older adults in urban areas, and older adults participating in medical insurance play a very important role in the medical security system. SMI can meet the basic medical needs of the urban older adults, CMI can effectively protect the personalized medical service needs of older adults, properly handle the relationship between SMI and CMI, can better play the role of the MIS. Second, we should not blindly pursue the coverage of SMI while ignoring the development of CMI. The government needs to continuously enrich and improve the supply of CMI products, encourage CMI companies to develop insurance types that are compatible with SMI and suitable for urban older adults groups, and pay attention to the medical service needs of urban older adults groups. Thirdly, medical insurance needs to take targeted measures according to the characteristics of the group if it wants to serve older adults better. We will comprehensively improve the level of medical service coverage for different groups, and make the content and level of medical insurance coverage more complementary and cohesive.

There are limitations to the study. Firstly, this paper focuses only on the health status of older adults aged sixty and above and the impact of medical insurance on their health, but not on the people below sixty. Although older adults are somewhat representative, they are not representative of all groups because of the heterogeneity of health status among different groups and the different impact of medical insurance. Future studies should expand the study to include more individuals. Secondly, we cannot account for the uninsured older adults. We estimate that some older adults who are not covered by medicare have health problems that are not diagnosed in a timely manner. This suggests that we may be underestimating medicare’s contribution to older adults’ health. If so, the effect of medical insurance may be much bigger than we estimate. Despite the limitations of the study, we believe that this study makes full use of vertical panel data and provides new insights for understanding the relationship between MIS and health. We also look forward to China’s experience in reforming the MIS, providing valuable lessons for countries around the world seeking to reform social insurance plans.

## Figures and Tables

**Table 1:** Descriptive statistics of the main variables.

Variables	Variable Description	Mean	Standard	Min	Max
Physical Health	Continuous variable	22.968	2.430	6.000	24.000
Mental Health	Continuous variable	33.614	5.451	10.000	40.000
Self-Health	Continuous variable	3.126	0.932	1.000	5.000
Social Medical Insurance (SMI)	With social medical insurance = 1	0.892	0.311	0.000	1.000
Commercial Medical Insurance (CMI)	With commercial medical insurance = 1	0.351	0.478	0.000	1.000
Gender	Male = 1	0.473	0.499	0.000	1.000
Age	Continuous variable	70.276	6.978	60.000	97.000
Marriage	Normal = 1	0.790	0.407	0.000	1.000
Education	Continuous variable	1.896	1.054	1.000	5.000
Pension insurance	With pension insurance = 1	0.757	0.429	0.000	1.000
Chronic	With Chronic = 1	0.276	0.447	0.000	1.000
Children number	Continuous variable	2.884	2.292	0.000	21.000
Health behavior	Have healthy behavior = 1	0.912	0.284	0.000	1.000

**Table 2:** Regression results of the impact of China’s MIS on the health of urban older adults.

Variables	Model 2-1			Model 2-2		
	Physical Health	Mental Health	Self-Health	Physical Health	Mental Health	Self-Health
SMI	0.072 (0.185)	0.069 (0.403)	0.064 (0.075)	0.072 (0.268)	0.986 (0.804)	0.054 (0.121)
CMI	0.121 (0.125)	0.081 (0.252)	0.043 (0.053)	0.123 (0.330)	0.034 (0.902)	0.101 (0.165)
Gender				−0.043 (0.207)	0.794 (0.586)	0.144 (0.092)
Age				−0.023 (0.015)	0.045 (0.049)	0.009 (0.007)
Marriage				0.122 (0.213)	1.092 * (0.628)	−0.044 (0.097)
Education				0.068 (0.094)	0.417 (0.265)	0.006 (0.042)
Pension insurance				0.015 (0.277)	0.090 (0.853)	0.112 (0.137)
Chronic				−0.415 ** (0.194)	−1.123 ** (0.557)	−0.355 *** (0.085)
Children number				−0.298 *** (0.046)	−0.329 ** (0.144)	−0.027 (0.023)
Health behavior				1.965 *** (0.296)	3.358 *** (0.977)	0.372 ** (0.144)
Constant				23.941 *** (1.203)	25.255 *** (3.846)	2.193 *** (0.559)

Note: t-values are reported in parentheses. ***, **, and * represent significance at the levels of 1%, 5%, and 10%, respectively. SMI stands for social medical insurance, CMI stands for commercial medical insurance.

**Table 3:** Regression results of the robustness test.

Variables	Model 3-1			Model 3-2		
	Physical Health	Mental Health	Self-Health	Physical Health	Mental Health	Self-Health
SMI	0.137 (0.273)	0.891 (0.815)	0.063 (0.123)	0.273 (0.627)	0.458 (1.273)	0.248 (0.275)
CMI	0.698 (0.526)	1.009 (1.585)	0.181 (0.239)	0.724 (0.794)	1.585 (1.537)	0.401 (0.363)
Gender	−0.027 (0.208)	0.948 (0.583)	0.146 (0.092)	−0.192 (0.452)	1.627 * (0.929)	−0.168 (0.191)
Age	−0.023 (0.015)	0.026 (0.049)	0.008 (0.007)	−0.117 *** (0.029)	−0.095 (0.064)	−0.002 (0.012)
Marriage	0.138 (0.213)	1.065 * (0.623)	−0.045 (0.098)	−1.032 * (0.588)	−0.617 (1.231)	−0.195 (0.244)
Education	0.069 (0.094)	0.375 (0.263)	0.004 (0.042)	0.093 (0.243)	1.092 ** (0.459)	0.090 (0.099)
Pension insurance	0.043 (0.278)	0.190 (0.857)	0.106 (0.138)	0.772 (0.489)	−0.871 (1.023)	0.158 (0.202)
Chronic	−0.379 * (0.197)	−0.903 (0.560)	−0.35 3 *** (0.087)	−1.009 (0.692)	−0.169 (1.189)	−0.166 (0.225)
Children number	−0.296 *** (0.047)	−0.249 * (0.146)	−0.023 (0.024)	0.052 (0.088)	−0.008 (0.215)	−0.014 (0.035)
Health behavior	2.011 *** (0.298)	3.625 *** (0.973)	0.379 ** (0.146)	2.766 *** (0.542)	3.312 ** (1.336)	0.076 ** (0.325)
Year fixed effect	√	√	√			
Province fixed effects	√	√	√			
Constant	24.949 *** (1.363)	28.899 *** (4.231)	2.133 *** (0.636)	29.092 *** (2.434)	39.431 *** (5.206)	3.552 *** (1.066)

Note: t-values are reported in parentheses. ***, **, and * represent significance at the levels of 1%, 5%, and 10%, respectively. SMI stands for social medical insurance, CMI stands for commercial medical insurance. The √ means “Yes”.

**Table 4:** Regional test results of sample data.

Variables	Older Adults Living in Eastern China			Older Adults Living in Central China			Older Adults Living in Western China		
	Physical Health	Mental Health	Self-Health	Physical Health	Mental Health	Self-Health	Physical Health	Mental Health	Self-Health
SMI	0.006 (0.383)	2.708 ** (1.118)	0.159 (0.189)	0.964 (0.532)	0.775 (1.659)	0.140 (0.246)	0.505 (0.467)	0.172 (1.543)	0.082 (0.212)
CMI	0.255 (0.501)	0.661 (1.464)	0.019 (0.254)	0.332 (0.604)	1.365 (1.568)	0.374 (0.322)	0.905 (0.615)	1.422 (1.787)	0.308 (0.309)
Gender	0.326 (0.292)	0.484 (0.853)	0.185 (0.145)	0.009 (0.404)	1.942 * (1.024)	0.161 (0.174)	−0.474 (0.372)	0.135 (1.210)	0.163 (0.167)
Age	−0.060 ** (0.025)	−0.023 (0.074)	0.013 (0.012)	−0.023 (0.031)	−0.050 (0.094)	0.006 (0.015)	−0.031 (0.027)	−0.009 (0.096)	−0.013 (0.013)
Marriage	−0.019 (0.288)	2.062 ** (0.882)	−0.096 (0.148)	0.083 (0.445)	−1.321 (1.211)	−0.115 (0.206)	0.276 (0.379)	1.864 (1.246)	0.048 (0.171)
Education	0.056 (0.129)	0.584 (0.374)	0.047 (0.065)	−0.006 (0.183)	0.303 (0.486)	−0.086 (0.081)	0.052 (0.178)	0.247 (0.545)	0.076 (0.078)
Pension insurance	0.208 (0.402)	0.497 (1.295)	−0.166 (0.213)	−0.114 (0.574)	0.889 (1.675)	0.138 (0.298)	0.217 (0.466)	−1.027 (1.541)	0.306 (0.219)
Chronic	−0.295 (0.271)	−1.839 ** (0.798)	−0.431 *** (0.135)	−0.787 ** (0.363)	−1.023 (0.998)	−0.359 ** (0.159)	−0.216 (0.366)	0.128 (1.125)	−0.220 (0.157)
Children number	0.030 (0.088)	0.364 (0.261)	0.018 (0.044)	−0.384 *** (0.096)	−0.198 (0.276)	−0.064 (0.045)	−0.333 *** (0.067)	−0.585 ** (0.236)	−0.013 (0.035)
Health behavior	1.619 *** (0.359)	3.318 ** (1.254)	0.497 (0.199)	2.565 *** (0.615)	1.333 (1.802)	0.069 (0.289)	2.231 *** (0.619)	7.399 *** (2.224)	0.776 ** (0.307)
Constant	25.909 (1.782)	27.271 *** (5.416)	1.829 ** (0.884)	24.654 *** (2.688)	35.385 *** (7.776)	3.166 ** (1.234)	23.809 *** (1.983)	26.444 *** (7.444)	2.912 *** (0.959)

Note: t-values are reported in parentheses. ***, **, and * represent significance at the levels of 1%, 5%, and 10%, respectively. SMI stands for social medical insurance, CMI stands for commercial medical insurance.

**Table 5:** Test results of sample data by age group.

Variables	Older Adults between Age 60 and 74			Older adults Aged 75 and Above		
	Physical Health	Mental Health	Self-Health	Physical Health	Mental Health	Self-Health
SMI	−0.169 (0.291)	0.660 (1.030)	−0.095 (0.163)	0.008 (0.468)	1.619 (1.309)	0.266 (0.175)
CMI	−0.423 (0.322)	−1.288 (1.050)	0.059 (0.207)	1.255 * (0.717)	4.898 ** (1.871)	0.289 * (0.158)
Gender	−0.208 (0.208)	0.888 (0.679)	0.087 (0.113)	0.332 (0.415)	0.759 (1.182)	0.260 (0.284)
Age	−0.009 (0.031)	0.142 (0.107)	0.017 (0.017)	−0.131 *** (0.041)	−0.106 (0.144)	0.026 (0.018)
Marriage	0.153 (0.224)	1.392 * (0.775)	0.125 (0.127)	0.131 (0.397)	0.429 (1.101)	−0.316 ** (0.149)
Education	0.109 (0.108)	0.609 * (0.347)	0.049 (0.058)	−0.104 (0.161)	0.177 (0.419)	−0.035 (0.059)
Pension insurance	−0.263 (0.286)	−0.224 (1.025)	0.157 (0.177)	0.953 * (0.553)	1.986 (1.659)	0.101 (0.221)
Chronic	−0.249 (0.202)	−1.498 ** (0.668)	−0.210 * (0.109)	−0.937 ** (0.364)	−0.301 (1.017)	−0.579 *** (0.135)
Children number	−0.169 *** (0.053)	−0.420 ** (0.189)	−0.044 (0.032)	−0.404 *** (0.075)	−0.192 (0.222)	0.004 (0.032)
Health behavior	1.432 *** (0.382)	2.849 ** (1.369)	0.161 (0.212)	1.989 *** (0.449)	4.118 *** (1.429)	0.593 *** (0.188)
Constant	23.268 *** (2.117)	19.709 ** (7.490)	1.738 (1.175)	32.662 *** (3.443)	33.815 *** (11.511)	0.599 (1.465)

Note: t-values are reported in parentheses. ***, **, and * represent significance at the levels of 1%, 5%, and 10%, respectively. SMI stands for social medical insurance, CMI stands for commercial medical insurance.

**Table 6:** Mediating effect of future life security in the effect of CMI on the health of older adults aged 75 and above.

Variables	Physical Health			Mental Health			Self-Health		
	Main	Mediating		Main	Mediating		Main	Mediating	
	Model 6-1	Model 6-2	Model 6-3	Model 6-4	Model 6-2	Model 6-5	Model 6-6	Model 6-2	Model 6-7
CMI	1.255 * (0.715)	0.705 * (0.409)	0.496 (0.699)	4.917 ** (1.874)	0.705 * (0.409)	3.459 ** (1.654)	0.342 ** (0.131)	0.705 * (0.409)	0.041 (0.308)
Future life security			0.242 ** (0.119)			2.229 *** (0.322)			0.139 *** (0.049)
Gender	0.333 (0.413)	0.142 (0.250)	0.441 (0.370)	0.834 (1.182)	0.142 (0.250)	0.529 (1.036)	0.229 (0.284)	0.142 (0.250)	0.238 (0.157)
Age	−0.131 *** (0.041)	−0.005 (0.029)	−0.113 *** (0.042)	−0.103 (0.145)	−0.005 (0.029)	−0.076 (0.127)	0.025 (0.018)	−0.005 (0.029)	0.027 (0.018)
Marriage	0.132 (0.396)	−0.212 (0.235)	0.036 (0.350)	0.444 (1.103)	−0.212 (0.235)	0.859 (0.967)	−0.312 ** (0.149)	−0.212 (0.235)	−0.309 ** (0.148)
Education	−0.104 (0.159)	0.177 * (0.091)	−0.105 (0.137)	0.098 (0.415)	0.177 * (0.091)	−0.244 (0.367)	−0.049 (0.058)	0.177* (0.091)	−0.071 (0.058)
Pension insurance	0.934 * (0.551)	0.325 (0.352)	0.445 (0.554)	2.014 (1.661)	0.325 (0.352)	1.533 (1.456)	0.129 (0.221)	0.325 (0.352)	0.011 (0.229)
Chronic	−0.937 ** (0.362)	0.264 (0.218)	0.771 ** (0.333)	−0.308 (1.019)	0.264 (0.218)	−1.186 (0.901)	−0.589 *** (0.135)	0.264 (0.218)	−0.543 *** (0.136)
Children number	−0.404 *** (0.074)	0.012 (0.048)	−0.122 (0.076)	−0.197 (0.223)	0.012 (0.048)	−0.219 (0.195)	0.001 (0.032)	0.012 (0.048)	−0.007 (0.032)
Health behavior	1.989 *** (0.446)	0.199 (0.295)	1.512 *** (0.424)	3.896 *** (1.421)	0.199 (0.295)	3.179 ** (1.248)	0.558 *** (0.187)	0.199 (0.295)	0.592 *** (0.188)
Constant	32.671 *** (3.386)	2.086 (2.341)	30.472 *** (3.432)	35.299 *** (11.468)	2.086 (2.341)	29.349 *** (10.076)	0.878 (1.459)	2.086 (2.341)	0.524 (1.479)

Note: t-values are reported in parentheses. ***, **, and * represent significance at the levels of 1%, 5%, and 10%, respectively. CMI stands for commercial medical insurance.

**Table 7:** Mediating effect of future life security in the effect of SMI on the mental health of older adults living in eastern China.

Variables	Main	Mediating	
	Model7-1	Model7-2	Model7-3
SMI	2.659 ** (1.110)	0.256 *** (0.092)	2.573 ** (0.964)
Future life security			2.034 *** (0.277)
Gender	0.483 (0.851)	0.047 (0.208)	0.457 (0.739)
Age	−0.019 (0.073)	0.001 (0.018)	−0.015 (0.064)
Marriage	2.048 ** (0.879)	0.135 (0.214)	1.766 ** (0.764)
Education	0.578 (0.373)	0.028 (0.273)	0.049 (0.332)
Pension insurance	0.596 (1.273)	0.492 (0.309)	−0.297 (1.112)
Chronic	−1.779 ** (0.785)	−0.051 (0.191)	−1.719 (0.682)
Children number	0.351 (0.258)	−0.046 (0.063)	0.449 ** (0.225)
Health behavior	3.329 *** (1.250)	0.200 (0.297)	2.834 *** (1.088)
Constant	26.901 *** (5.341)	1.707 (1.273)	22.981 *** (4.669)

Note: t-values are reported in parentheses. *** and ** represent significance at the levels of 1%, 5% respectively. SMI stands for social medical insurance.

## Data Availability

The data presented in this study are available on request from the corresponding author. The data are not publicly available due to confidentiality.
